# Modeling heterogeneity of diabetic foot self-care behaviors in Al Qassim Region in Saudi Arabia

**DOI:** 10.25122/jml-2025-0137

**Published:** 2025-09

**Authors:** Reem Alsalamah

**Affiliations:** 1 Surgery Department, College of Medicine, Qassim University, Saudia Arabia

**Keywords:** diabetic foot, diabetes, diabetic complications, structural equation modelling, educational interventions

## Abstract

Diabetic foot complications are a major cause of morbidity and mortality, particularly in Saudi Arabia. Understanding how knowledge influences preventive practices is critical for designing effective interventions. A cross-sectional study was conducted among 647 diabetic patients in Al-Qassim, utilizing self-administered questionnaires to assess their knowledge, attitudes, and practices regarding diabetic foot care. Confirmatory factor analysis identified three knowledge domains (physiological, complication, preventive), two attitude constructs, and four practice domains. Structural equation modeling was employed to compare direct, full, and partial mediation models, with multi-group analysis used to assess demographic moderators. Among participants, 74.2% demonstrated good knowledge and 93.4% reported positive attitudes, but only 63.7% had adequate practices. The partial mediation model showed the best fit (CFI = 0.938, RMSEA = 0.049), with 59.8% of knowledge effects on practices mediated through attitudes. Preventive knowledge exerted the strongest effects on attitudes (β = 0.497, *P* < 0.001) and practices (β = 0.482, *P* < 0.001), while physiological knowledge had no direct impact. Knowledge-practice pathways were significantly stronger in patients with higher education, longer diabetes duration, and greater exposure to formal education. Attitudes primarily mediate the link between knowledge and practice. Effective interventions should emphasize preventive knowledge, address attitudinal barriers, and be tailored to demographic profiles. Achieving at least 70% knowledge mastery appears essential for improving preventive behaviors.

## Introduction

Diabetes mellitus represents one of the most significant global health challenges of the 21^st^ century, with an increasing prevalence rate predicted to affect around 700 million adults all over the world by 2045 [1]. Among the several complications associated with diabetes, diabetic foot associations remain a burden, often leading to lower extremity amputations that majorly impact patients' quality of life, functional independence, and even survival outcomes [1]. The pathophysiology of diabetic foot complications originates from an interplay of peripheral neuropathy, peripheral arterial disease, altered biomechanics, and susceptibility to infection. These factors together form a high-risk environment where even minor trauma can lead to events resulting in ulceration, disease, and limb loss [2].

The economic burden of diabetic foot disease is of significant interest, with medical costs estimated to be between $9 billion and $ 13 billion each year in the United States of America alone, beyond the standard costs of diabetes management [3,4]. In Saudi Arabia, where diabetes prevalence ranks among the highest globally, affecting around 18.3% of the adult population, diabetic foot complications are a significant healthcare challenge. Previous studies have shown that Saudi patients exhibit varying levels of knowledge about diabetes management; however, specific investigations into the relationships between knowledge, attitudes, and practices (KAP) regarding diabetic foot care remain limited [5].

While previous studies have demonstrated knowledge deficits or inadequate foot care practices among diabetic populations, few have investigated the structural pathways through which knowledge affects actual preventive behaviors. Previous Saudi-based studies have primarily examined the correlational relationships between knowledge scores and practice measures, without elucidating the psychological mechanisms that mediate this relationship, which creates a gap in understanding how educational interventions should be designed and applied more effectively [6–10].

The concept that knowledge alone is insufficient to change behavior is well-established in health psychology, with theoretical frameworks such as the KAP model suggesting that attitudes serve as an important mediating factor. However, the specific pathways and relative strengths of these relationships in diabetic foot care remain poorly demarcated in current literature evidence. Structural equation modeling (SEM) offers an analytical approach that enables the simultaneous examination of multiple relationships between latent constructs, while also accounting for measurement error, making it a suitable choice for investigating these factors and pathways [11].

To my knowledge, no Saudi study has used SEM to disentangle the mediating role of attitudes in diabetic foot care. This analytical approach enables us to move beyond simple correlations and reveal the mechanisms that underlie preventive behaviors, making both conceptual and practical contributions to diabetic education in Saudi Arabia.

Therefore, this study aims to utilize SEM to identify the direct and indirect pathways through which specific domains of diabetic foot knowledge impact the preventive practices among patients in the Al-Qassim region, Saudi Arabia. We aimed to: (1) determine the factor structure of KAP domains; (2) assess the direct and indirect effects of knowledge on practice, with attitudes as possible mediators; (3) identify demographic and clinical factors that moderate these relationships; and (4) determine high-leverage intervention points to inform more effective educational strategies.

## Material and Methods

### Study design and population

A cross-sectional, questionnaire-based study was conducted among patients with diabetes mellitus residing in the Al-Qassim region of Saudi Arabia between May 2024 and October 2024. The study utilized a descriptive, analytical approach to evaluate the KAP regarding diabetic foot care. According to the latest statistics from the General Authority for Statistics in Saudi Arabia, Al-Qassim has a population of around 1,488,285 inhabitants. We determined our sample size using the Raosoft Sample Size Calculator, with a 5% margin of error and a 95% confidence level, yielding a target sample size of 377 participants. To further validate the adequate statistical power for our SEM analyses, we aimed to recruit at least 600 participants.

### Participant selection and recruitment

Individuals aged 18 years and above residing in the Al-Qassim region, diagnosed with either type 1 or type 2 diabetes mellitus, were eligible for inclusion. Participants were recruited through multiple channels, including primary healthcare centers, diabetes clinics, and social media platforms such as WhatsApp, X (formerly Twitter), and Instagram. Before participation, each individual provided electronic informed consent. Electronic informed consent was obtained from all participants prior to enrollment. Data were collected using a self-administered online questionnaire distributed via Google Forms. To prevent incomplete submissions, all items were set as mandatory. The questionnaire was made easily accessible, and assistance was provided to participants when needed throughout the data collection period.

### Instruments and measures

The questionnaire has four main sections: demographic information, knowledge assessment, attitude assessment, and practice assessment. The demographic section collected information about participants' gender, age, nationality, marital status, education level, occupation, income, duration of diabetes, and frequency of clinic visits. The knowledge assessment section included 12 key questions covering various aspects of diabetic foot care, including physiological mechanisms, possible complications, and preventive measures. Responses were recorded on a five-point Likert scale ranging from 'strongly agree' to 'strongly disagree'. The attitude assessment included five questions addressing participants' perspectives on nutrition, footwear, healthcare utilization, exercise, and education regarding diabetic foot care. The practice assessment section consisted of 16 questions regarding specific foot care behaviors, including hygiene practices, inspection practices, protective measures, and healthcare utilization.

### Knowledge classification and scoring

To assess participants' knowledge about foot care, we included 12 key questions in the questionnaire. Each correct or appropriate response indicating accurate knowledge was awarded one point, while incorrect or 'unsure' responses received zero points, resulting in a maximum possible score of 12. Based on the total score, participants were classified into two categories: those who scored eight or more points (≥66.7%) were considered to have good knowledge, while those who scored seven or fewer points (<66.7%) were categorized as having poor knowledge.

### Data analysis and structural equation modeling

All analyses were performed in RStudio using R version 4.4.2, following a five-phase analytical process. In the first phase, data cleaning and preliminary diagnostics were conducted, including assessment of multivariate normality and treatment of missing data using multiple imputation techniques. In the second phase, the measurement model was specified and validated using confirmatory factor analysis (CFA). The model incorporated three knowledge constructs (physiological knowledge, complication knowledge, and preventive knowledge), two attitude constructs (treatment adherence and preventive behavior), and four practice constructs (hygiene practices, inspection practices, protective practices, and healthcare utilization).

**Figure 1:**
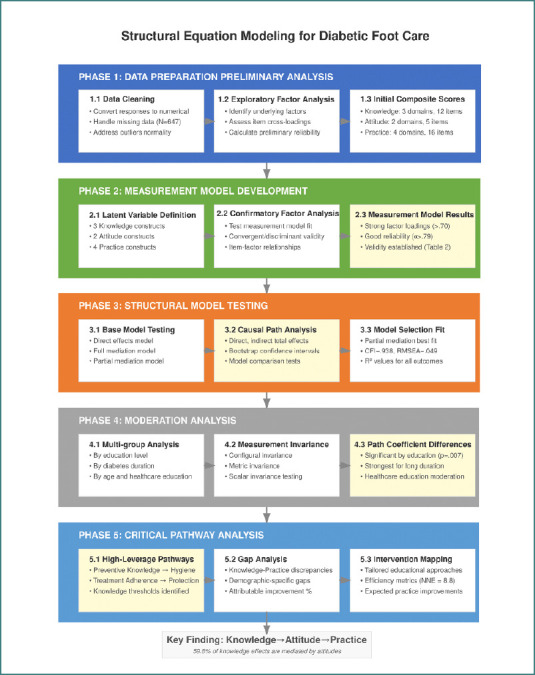
Structural equation modelling process flowchart

The third phase involved testing the structural model under three specifications: a direct effects model (knowledge to practice), a full mediation model (knowledge to attitude to practice), and a partial mediation model with both direct and indirect paths. Standardized path coefficients, direct effects, indirect effects, and total effects were calculated for all pathways. Statistical significance was evaluated using bootstrapped confidence intervals based on 5,000 resamples. In the fourth phase, moderation analyses were conducted using multi-group structural equation modeling (SEM) to determine whether pathway strengths differed by education level, duration of diabetes, exposure to healthcare provider education, and age group. Measurement invariance was tested prior to comparing structural paths across groups. In the final phase, critical pathway analysis was performed to identify high-impact knowledge components, threshold effects in knowledge-to-practice translation, demographic-specific gaps, and the proportion of practice improvements attributable to specific constructs. All statistical tests were two-tailed with a significance level of 0.05 (Figure 1).

## Results

### Participants and KAP characteristics

Table 1 presents the characteristics of our study participants. Among the 647 diabetic patients, slightly more than half were men (55.0%), with nearly one-third (30.0%) aged between 18 and 29 years. The majority were Saudi nationals (93.8%), and almost three-quarters (73.9%) had attained university education or higher. The government sector represented the most common occupation (32.3%), while more than one-third (37.1%) reported a monthly income of less than 5,000 Saudi riyals (SAR). Regarding diabetes-related characteristics, a significant proportion (62.8%) had a family history of diabetes, and more than one-third (37.9%) reported fewer than two visits per year. Of special concern, 29.1% of participants had experienced foot sores or wounds, with 42.6% of these cases being self-treated rather than receiving professional medical attention. In terms of KAP scores, nearly three-quarters (74.2%) demonstrated good knowledge about foot care, while most of the participants (93.4%) showed positive attitudes. For specific practice domains, our findings revealed varying levels of adherence, with hygiene practices showing the highest adherence (86.9% washing feet daily) and inspection practices showing the lowest adherence (only 31.5% using mirrors to check foot soles).

**Table 1:** Patients’ characteristics and metrics (*n* = 647)

Characteristic	Category	Number (%) or Mean ± SD
**Sociodemographic characteristics**		
Sex	Men	356 (55.0)
	Women	291 (45.0)
Age (years)	Under 18 years	17 (2.6)
	18-29	194 (30.0)
	30-39	101 (15.6)
	40-49	142 (21.9)
	50-59	118 (18.2)
	60 years and above	75 (11.6)
Nationality	Saudi	607 (93.8)
	Non-Saudi	40 (6.2)
Marital status	Single	223 (34.5)
	Married	399 (61.7)
	Divorced	12 (1.9)
	Widowed	13 (2.0)
Education level	Primary education and below	14 (2.2)
	Intermediate education	29 (4.5)
	Secondary education	126 (19.5)
	University education or higher	478 (73.9)
Profession	Government sector employee	209 (32.3)
	Private sector employee	95 (14.7)
	Retired	116 (17.9)
	Student	132 (20.4)
	Unemployed	95 (14.7)
Monthly income (SAR)	Less than 5000	240 (37.1)
	5000-10000	132 (20.4)
	10000-15000	111 (17.2)
	More than 15000	164 (25.3)
**Clinical characteristics**		
Duration of diabetes (years)	Less than a year	27 (4.2)
	1-5 years	65 (10.0)
	6-10 years	52 (8.0)
	More than 10 years	97 (15.0)
	Family history of diabetes	406 (62.8)
Number of clinic visits per year	Less than two visits	245 (37.9)
	2-4 visits	229 (35.4)
	5-7 visits	102 (15.8)
	More than 7 visits	71 (11.0)
History of foot sores/wounds	Yes	188 (29.1)
	No	459 (70.9)
Management of foot sores/wounds	Sought medical attention	108 (57.4)
	Self-treated	80 (42.6)
**Knowledge, attitude, and practice metrics**		
Knowledge score	Mean total score (range: 12-60)	44.3 ± 6.2
	Good knowledge (≥8 points, ≥66.7%)	480 (74.2)
	Poor knowledge (<8 points, <66.7%)	167 (25.8)
	Highest: Diabetics develop gangrene in the foot	590 (91.2)
	Lowest: Signs of diabetic foot infection	393 (60.7)
Attitude score	Mean total score (range: 0-5)	4.2 ± 0.8
	Positive attitude	604 (93.4)
	Poor attitude	43 (6.6)
	Highest: Nutrition is important for blood sugar	615 (95.1)
	Lowest: Received foot care education	327 (50.5)
Practice score	Mean total score (range: 0-16)	9.6 ± 3.5
	Good practices	412 (63.7)
	Poor practices	235 (36.3)
	Highest: Washing feet daily	562 (86.9)
	Lowest: Using a mirror to check foot soles	1.5)

### Measurement model and factor structure

**Figure 2:**
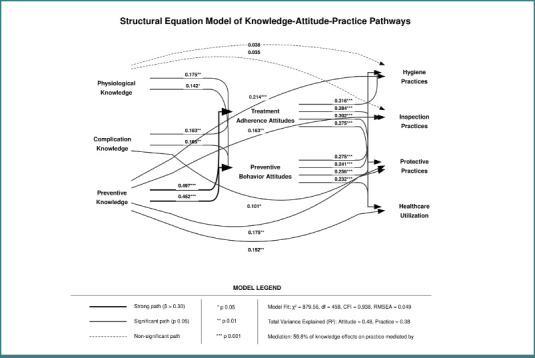
Structural equation model diagram

As shown in Table 2, our measurement model demonstrated excellent psychometric properties across all latent constructs. The confirmatory factor analysis findings have supported our hypothesized three-factor structure for knowledge (physiological, complication, and preventive knowledge), a two-factor structure for attitudes (treatment adherence and preventive behavior), and a four-factor structure for practices (hygiene, inspection, protective, and healthcare utilization). All factor loadings exceeded the recommended threshold of 0.70, which reflects strong item-factor relationships. Reliability coefficients were significant across all constructs, with Cronbach's alpha values ranging from 0.79 to 0.89, composite reliability values from 0.81 to 0.90, and average variance extracted values from 0.59 to 0.70, collectively indicating excellent internal consistency and convergent validity. The square roots of AVE exceeded inter-construct correlations for all pairs of factors, confirming discriminant validity. Overall model fit indices (CFI = 0.942, TLI = 0.935, RMSEA = 0.048, SRMR = 0.057) demonstrated that our measurement model provided a well-performing representation of the underlying factor structure (Figure 2).

**Table 2:** Factor structure, psychometric properties, and discriminant validity of knowledge, attitude, and practice constructs

Construct/Items	Factor Loading (Std.)	Item-Total Correlation	Communality (h^2^)	Reliability (α, AVE, CR) / Fit
**Knowledge Construct 1: Physiological Knowledge**				**α = 0.84**, **AVE = 0.62**, **CR = 0.87**
K1: Reduced blood flow	0.78	0.70	0.61	
K2: Loss of sensation	0.85	0.77	0.72	
K8: High blood sugar damages vessels	0.77	0.69	0.59	
K7: Changes in foot shape	0.75	0.67	0.56	
**Knowledge Construct 2: Complication Knowledge**				**α = 0.89**, **AVE = 0.70**, **CR = 0.90**
K3: Lead to foot ulcers	0.88	0.82	0.77	
K4: Develop gangrene	0.87	0.80	0.76	
K5: Loss of sensation -> ulcers	0.84	0.76	0.71	
K6: Decreased blood flow -> ulcers	0.83	0.75	0.69	
K9: Signs of infection	0.72	0.63	0.52	
**Knowledge Construct 3: Preventive Knowledge**				**α = 0.82**, **AVE = 0.61**, **CR = 0.83**
K10: Proper shoes	0.82	0.74	0.67	
K11: Smoking increases complications	0.75	0.66	0.56	
K12: Regular foot care prevents problems	0.86	0.79	0.74	
**Attitude Construct 1: Treatment Adherence Attitudes**				**α = 0.83**, **AVE = 0.63**, **CR = 0.84**
A1: Nutrition important	0.77	0.68	0.59	
A2: Willing to use special shoes	0.81	0.73	0.66	
A3: Regular podiatrist visits necessary	0.83	0.75	0.69	
**Attitude Construct 2: Preventive Behavior Attitudes**				**α = 0.79**, **AVE = 0.65**, **CR = 0.81**
A4: Exercise helps foot health	0.84	0.76	0.71	
A5: Received education	0.77	0.68	0.59	
**Practice Construct 1: Hygiene Practices**				**α = 0.86**, **AVE = 0.64**, **CR = 0.88**
P_Wash: Wash daily	0.85	0.77	0.72	
P_Dry: Dry between toes	0.81	0.72	0.66	
P_Lotion: Use lotion	0.75	0.65	0.56	
P_Nails: Cut nails straight	0.79	0.70	0.62	
**Practice Construct 2: Inspection Practices**				**α = 0.83**, **AVE = 0.62**, **CR = 0.85**
P_CheckDaily: Check daily	0.84	0.76	0.71	
P_Mirror: Use mirror for soles	0.77	0.67	0.59	
P_CheckShoes: Check shoes before wearing	0.80	0.71	0.64	
**Practice Construct 3: Protective Practices**				**α = 0.85**, **AVE = 0.61**, **CR = 0.86**
P_Socks: Wear diabetic socks	0.76	0.67	0.58	
P_Barefoot: Avoid barefoot	0.83	0.75	0.69	
P_ProtectTemp: Protect from temp extremes	0.79	0.70	0.62	
P_HeatPads: Avoid heating pads	0.75	0.66	0.56	
P_SharpTools: Avoid harsh chemicals/tools	0.78	0.69	0.61	
**Practice Construct 4: Healthcare Utilization**				**α = 0.80**, **AVE = 0.59**, **CR = 0.81**
P_Sores: Sought medical attention for sores	0.78	0.69	0.61	
P_Sugar: Monitor blood sugar	0.81	0.72	0.66	
P_Activity: Regular exercise	0.72	0.63	0.52	
**Confirmatory Factor Analysis (CFA) Model Fit Indices**				
Chi-Square (χ^2^)	863.42			
Degrees of Freedom (df)	467			
χ^2^/df ratio	1.85			
P-value	< 0.001			
Comparative Fit Index (CFI)	0.942			
Tucker-Lewis Index (TLI)	0.935			
Root Mean Square Error of Approximation (RMSEA)	0.048			
90% RMSEA Confidence Interval	[0.043, 0.053]			
Standardized Root Mean Square Residual (SRMR)	0.057			

Notes and Abbreviations: *factor loadings (Std. = Standardized), item-total correlations, communality (h^2^), Cronbach's alpha (α), Average Variance Extracted (AVE), and Composite Reliability (CR). CFA Model Fit based on Confirmatory Factor Analysis. Table 2 shows Discriminant Validity via Fornell-Larcker criterion: Diagonal values (bold) are the square root of AVE for each construct and should exceed inter-construct correlations (off-diagonal values) for adequate discriminant validity.

### Structural pathways and mediation effects

**Figure 3:**
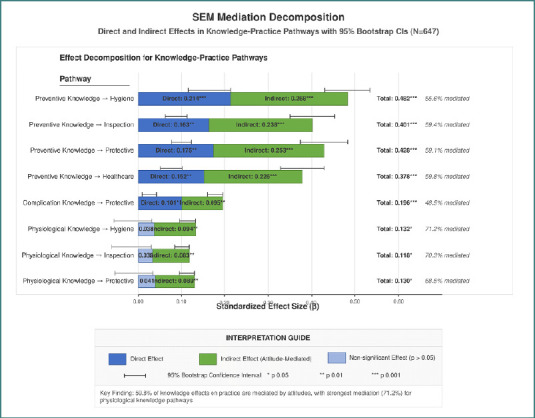
Structural equation model mediation decomposition

Supplementary Table 1 presents the direct, indirect, and total effects in the KAP pathways. Our analysis of competing models revealed that the partial mediation model demonstrated significantly better fit (χ^2^ = 879.56, df = 459, CFI = 0.938, RMSEA = 0.049) compared to both the direct effects only model (Δχ^2^ = 252.54, *P* value < 0.001) and the complete mediation model (Δχ^2^ = 115.58, *P* value < 0.001). The assessment of direct effects revealed that preventive knowledge had the most substantial impact on both attitude domains (β = 0.497 and β = 0.452, *P* < 0.001). In contrast, physiological and complication knowledge showed significant but weaker effects (β ranging from 0.142 to 0.183, *P* value < 0.05). In the attitude-to-practice pathways, treatment adherence attitudes demonstrated medium-strength effects on hygiene and protective practices (β = 0.316 and β = 0.302, *P* value < 0.001), with slightly smaller effects on inspection and healthcare utilization (β = 0.284 and β = 0.275, *P* value < 0.001). Regarding direct knowledge-to-practice effects, preventive knowledge maintained significant direct effects on all practice domains (β ranging from 0.152 to 0.214, *P* value < 0.01). In contrast, the physiological knowledge showed no significant direct effects. Indirect effect analysis revealed that 59.8% of knowledge effects on practice were mediated by attitudes, with preventive knowledge showing the most substantial indirect effects through attitudes to hygiene practices (β = 0.268, *P* value < 0.001). The total effects (direct plus indirect) were predominantly strong for preventive knowledge of hygiene practices (β = 0.482, *P* < 0.001) and protective practices (β = 0.428, *P* < 0.001; Figure 3).

### Moderation effects based on participants' characteristics

The results of our moderation analyses, presented in Supplementary Table 2, showed significant differences in KAP pathways across both demographic and clinical subgroups. Multi-group analyses indicated significant path invariance by education level (Δχ^2^ = 27.41, df = 12, *P* value = 0.007), diabetes duration (Δχ^2^ = 35.62, df = 12, *P* value < 0.001), healthcare education (Δχ^2^ = 41.83, df = 12, *P* value < 0.001), and age group (Δχ^2^ = 29.07, df = 12, *P* value = 0.004). Patients with higher education showed stronger pathways from preventive knowledge to treatment adherence attitudes (β = 0.512 vs. β = 0.462) and from complication knowledge to protective practices (β = 0.114 vs. β = 0.063). Similarly, those with longer diabetes duration exceeding ten years demonstrated significantly stronger pathways across all knowledge-attitude relationships compared to those with shorter durations, with the largest difference observed in the preventive knowledge to treatment adherence pathway (β = 0.583 vs. β = 0.426). Patients who received healthcare provider education about diabetic foot care showed consistently stronger pathways throughout the model, which were mostly in the knowledge-to-attitude relationships. Measurement invariance testing supported configural and metric invariance across all groups; however, scalar invariance was only partially supported for diabetes duration and healthcare education groups, suggesting some differences in intercepts across these groups.

### Critical pathways and intervention points

Supplementary Table 3 presents our critical pathway findings, which identified high-leverage knowledge components and practice outcomes for targeted interventions. The knowledge item with the highest total effect on practice outcomes was "regular foot care prevents problems" (β = 0.482, *P* value < 0.001), followed by "importance of wearing proper shoes" (β = 0.463, *P* value < 0.001). We identified significant knowledge threshold effects, with patients scoring above 42/60 (70%) on knowledge demonstrating a much higher probability of engaging in good hygiene practices (83.5% vs. 36.7%, OR = 8.7). The knowledge-practice gap findings revealed multiple differences across demographic segments, with younger patients under 40 years old showing the largest gap in inspection practices (31.4%) and patients without healthcare education showing gaps across all domains of over 20%. Our simulation of practice improvement with targeted knowledge improvements suggested that a structured and focused KAP intervention could improve the overall practice adherence by 18.6%. In comparison, a knowledge deficit identification approach would have only a 5.2% improvement. The critical mediation pathway results indicated that the preventive knowledge, followed by treatment adherence, followed by hygiene pathway had the highest mediation proportion, at a rate of 55.6%, which suggests that interventions focused on attitude formation regarding treatment adherence would have the most significant possible impact on the outcomes.

## Discussion

Diabetic foot complications are among the most devastating issues in diabetic patients, which could result in lower limb amputations, reduced quality of life, and increased mortality rates if not appropriately managed [12]. In Saudi Arabia, where diabetes prevalence ranks among the highest around the world, understanding the pathways through which patients acquire and implement foot care knowledge is of significant importance for developing effective preventive strategies [13,14]. Despite previous studies from Saudi Arabia on knowledge gaps in diabetic populations, the mechanisms by which knowledge impacts and affects actual preventive behaviors remain limited, especially in regions like Al-Qassim [6–10].

The findings from Al-Qassim revealed that preventive knowledge was the strongest driver of both attitudes and practices, with nearly 60% of the knowledge effect mediated through attitudes. This aligns closely with the study by Xie *et al*., where knowledge also showed a strong direct effect on attitudes (β = 0.75) and practices (β = 0.38), underscoring the universal role of preventive understanding in shaping self-care [15]. However, while Chinese patients reported low engagement in exercise and a predominance of rural residence, our Saudi sample was highly educated, suggesting different demographic moderators of the knowledge–practice link. The systematic review by Untari *et al*. confirmed global variability, with some studies reporting up to 88% of patients demonstrating good knowledge, while others found as many as 84.8% with poor knowledge, and practice most often limited to basic hygiene rather than inspection or footwear use [16]; the study adds novel evidence by quantifying a knowledge threshold (≥70%) necessary before practices improved significantly. In contrast, the qualitative study by Simonsen *et al*. highlighted how health literacy and cognitive function stratify patients into proactive, active, or passive profiles, while our quantitative model demonstrated how attitudes mediate practice gaps, offering complementary but convergent insights [17]. The large Turkish cohort reported by Karadağ *et al*. found that only 20.8% of patients achieved “good” foot care practices, a rate significantly lower than our 63.7%. This suggests that Saudi patients may benefit from higher educational levels or greater engagement with the health system. However, the Turkish data confirmed that education status and disease duration were key predictors, consistent with our moderation analyses, which showed stronger pathways among highly educated and longer-duration patients [18]. Together, these international comparisons demonstrate that while the knowledge–practice gap is global, the specific thresholds, mediators, and demographic moderators vary across settings. For Saudi Arabia, our findings uniquely show that even with high education levels, self-treatment of wounds (42.6%) remains a major risk, indicating a cultural preference for family-based care rather than professional consultation.

The KAP model provides a conceptual framework for understanding health behaviors, suggesting that knowledge acquisition leads to attitudinal changes, which in turn influence practices. However, the relative contributions of direct knowledge effects versus attitude-mediated pathways in diabetic foot care behaviors have not been previously studied or investigated using focused analytical methodologies, such as SEM, to date [19]. This study aimed to address this gap by providing a detailed analysis of these pathways among a large sample of diabetic patients in the Al-Qassim region of Saudi Arabia.

The study's findings reveal that nearly three-quarters of participants demonstrated a good understanding of diabetic foot care, with a majority holding positive attitudes. However, this positive attitude did not consistently translate into adequate foot care practices, with significant gaps observed in essential behaviors such as daily foot inspection and proper footwear use. Through the SEM, we identified that the relationship between knowledge and practice is primarily mediated by attitudes, with 59.8% of knowledge effects on practice occurring indirectly through attitudinal changes rather than directly affecting behavior.

Among knowledge domains, preventive knowledge showed the most substantial impact on both attitudes and practices, with significant effects on hygiene practices. An interesting concern is that the physiological knowledge about understanding blood flow reduction, as well as sensation loss, showed no substantial direct effects on practice behaviors, despite being well understood by participants. In this study, moderation analyses revealed that knowledge-practice pathways differed significantly across demographic groups, with stronger knowledge-attitude-practice links observed in patients with higher education, longer diabetes duration, and those who had received formal education about foot care from healthcare providers. Critical pathway analysis identified specific knowledge thresholds, indicating that patients needed to achieve 70% of maximum knowledge scores before significant practice improvements occurred.

The identification of partial mediation as the best-fitting model has profound implications for clinical education and intervention design. Unlike previous studies that have focused chiefly on knowledge deficits, our findings suggest that simply providing information without addressing attitudes may result in only modest behavioral changes [6–10]. This explains the frequent observation of patients who can correctly recite foot care guidelines but fail to consistently implement them.

The strong pathway from preventive knowledge to treatment adherence, as well as attitudes towards hygiene practices, represents a critical intervention point for physicians. This finding suggests that education focusing specifically on preventive measures, including regular foot care, proper footwear, and smoking cessation, rather than on physiological mechanisms, may lead to better behavior change. This contrasts with typical diabetes education programs, which often focus on teaching and explaining pathophysiological concepts to individuals [20]. The results suggest that educational approaches should be restructured to focus on practical prevention strategies.

The moderation analysis highlighted important findings for practice, as the stronger KAP pathways among patients who had received formal education about foot care underscore the importance of structured educational programs. This finding suggests that healthcare providers should prioritize formal diabetic foot education, especially for newly diagnosed patients and those with lower educational levels, who demonstrated weaker knowledge-practice links. Similarly, the identification of specific knowledge thresholds suggests that educators should aim for detailed and comprehensive knowledge coverage rather than focusing on selective topics, as practice improvements accelerated significantly once patients reached 70% mastery of the knowledge.

The observed demographic variations in knowledge-practice gaps offer guidance for tailoring interventions. For instance, patients younger than 40 years old demonstrated certain gaps in inspection practices, which could reflect that this age group might benefit from a specific focus on daily foot examination routines, possibly leveraging technology or reminder systems that resonate with younger populations. Healthcare utilization appeared as a significant gap area for patients with longer diabetes duration, highlighting the importance of maintaining regular medical follow-up even among experienced patients who might feel confident in self-management. In this study, critical pathway analysis provided concrete guidance for prioritizing educational content. The finding that knowledge about regular foot care and proper footwear showed the highest total effects on practice outcomes suggests that these topics should form the core of diabetic foot education [21–23].

The findings point to several Saudi-specific strategies. First, routine diabetic foot education should be embedded into primary healthcare center visits, ensuring that physicians and nurses consistently reinforce preventive practices. Second, given that 42.6% of the patients reported self-treating wounds, community-based awareness campaigns through mosques and local leaders are essential to counter reliance on informal care. Third, younger patients under 40, who showed the weakest inspection practices, may benefit from mobile health applications or SMS reminders that promote daily foot checks. Fourth, diabetic foot education should be explicitly aligned with Saudi Vision 2030 health transformation goals, with knowledge mastery (≥70%) serving as a measurable quality indicator. Ultimately, cost-effectiveness analyses support subsidizing preventive footwear and podiatry consultations, which may help offset the high downstream costs associated with amputations. Together, these strategies move beyond generic calls for education by providing culturally and system-specific recommendations tailored to Saudi Arabia’s healthcare landscape.

Several limitations should be considered when interpreting our findings. First, the cross-sectional design of our study and data collection methods preclude the establishment of definitive causal relationships. While SEM methodologies allow us to test causal hypotheses [24], longitudinal data would provide stronger evidence for the temporal and time-changing sequences of knowledge acquisition, attitude formation, and real-world practice implementation. Resource constraints and the practical challenges of longitudinal follow-up in our region necessitated the use of a cross-sectional approach.

Second, this study's convenience sampling approach may have introduced selection bias, which could have overrepresented patients with higher education levels and greater healthcare engagement. The high proportion of university-educated participants, with the percentage of 73.9% exceeds the general population distribution in Al-Qassim, likely reflecting differential access to and comfort with online survey participation. This sampling limitation resulted from pandemic-related restrictions on in-person recruitment and the logistical challenges of probability sampling across the geographically dispersed region. Interpretation of results should therefore be made with caution, particularly when applying them to populations with lower educational attainment.

Third, this study's reliance on self-reported practices rather than direct observation may have introduced social desirability bias, with participants possibly overreporting adherence to recommended behaviors. While objective measurement of foot care practices would be ideal, the private nature of these behaviors and practical constraints made self-reporting the only feasible approach. While we attempted to mitigate these by using specific behavioral items rather than general adherence questions, the possibility of overreporting favorable practices remains.

Fourth, although the sample size was statistically adequate for the SEM analysis, some demographic subgroups had relatively small representations, which may have limited the power of our moderation analyses. This primarily affects the interpretation of findings related to patients with shorter diabetes duration and those with lower educational levels. We should also note that our study was conducted in a single region within Saudi Arabia, which may limit its generalizability to other areas with different healthcare systems, cultural practices, and considerations, as well as diabetes education programs.

Based on our findings and limitations, we recommend several directions for future studies and practice. First, longitudinal studies investigating the temporal sequence of knowledge acquisition, attitude formation, and practice implementation would provide stronger causal evidence. These could identify significant time points for intervention during the diabetes care journey. Such studies should integrate and include objective measures of foot care practices where feasible, perhaps through innovative strategies such as smart footwear technology or photographic documentation of foot conditions. Second, intervention studies specifically targeting the identified high-leverage pathways should be developed and tested. These interventions should be tailored to demographic subgroups based on our moderation findings, with a focus on younger patients, those with lower educational levels, and individuals newly diagnosed with the condition. Effectiveness should be evaluated not only through knowledge assessments but also through attitudinal measures and objective practice outcomes.

Third, we recommend that healthcare systems develop and implement knowledge threshold assessment tools based on our findings. Patients scoring below the identified thresholds with 70% on the knowledge measure should receive special, dedicated education focused on preventive topics rather than physiological mechanisms. This strategy would optimize resource allocation by concentrating educational efforts where they are most likely to result in practice improvements. Fourth, better-designed educational programs should be developed that explicitly address attitudinal barriers in addition to knowledge provision. These programs should include motivational elements, address perceived barriers to foot care practices, and build self-efficacy for specific behaviors, rather than focusing exclusively on knowledge transfer [25]. Such programs should be evaluated through randomized controlled trials comparing attitude-focused versus knowledge-only approaches.

Future studies should investigate the potential of digital health interventions aimed at enhancing the KAP pathways identified in our research. Mobile applications could be developed that not only provide knowledge but also integrate behavioral economics principles to address the attitudinal barriers and facilitate practice implementation using reminders, monitoring, and social support features. Such approaches may be especially practical for younger patients and those with barriers to regular healthcare access.

### Moderation effects based on the participants' characteristics

The results of the moderation analyses in this study, presented in Supplementary Table 2, showed significant differences in KAP pathways across both demographic and clinical subgroups. Multi-group analyses indicated significant path invariance by education level (Δχ^2^ = 27.41, df = 12, *P* value = 0.007), diabetes duration (Δχ^2^ = 35.62, df = 12, *P* value < 0.001), healthcare education (Δχ^2^ = 41.83, df = 12, *P* value < 0.001), and age group (Δχ^2^ = 29.07, df = 12, *P* value = 0.004). Patients with higher education showed stronger pathways from preventive knowledge to treatment adherence attitudes (β = 0.512 vs. β = 0.462) and from complication knowledge to protective practices (β = 0.114 vs. β = 0.063). Similarly, those with longer diabetes duration exceeding ten years have demonstrated significantly stronger pathways across all knowledge-attitude relationships compared to those with shorter durations, with the largest difference observed in the preventive knowledge to treatment adherence pathway (β = 0.583 vs. β = 0.426). Patients who had received education from a healthcare provider about diabetic foot care showed consistently stronger pathways throughout the model, which were mostly evident in the knowledge-to-attitude relationships. Measurement invariance testing supported configural and metric invariance across all groups; however, scalar invariance was only partially supported for diabetes duration and healthcare education groups, suggesting some differences in intercepts across these groups.

## Conclusion

The SEM results in this study demonstrate that the relationship between diabetic foot knowledge and preventive practices is primarily mediated through attitudes, with nearly 60% of knowledge effects occurring through this indirect pathway. Among knowledge domains, preventive knowledge had the greatest impact on both attitudes and practices, while physiological knowledge showed minimal direct effects, despite being well understood by participants. Knowledge threshold effects revealed that patients needed to achieve 70% of maximum knowledge scores before significant practice improvements occurred, with significantly stronger KAP pathways among patients with higher education, longer diabetes duration, and those who had received formal diabetic foot education from healthcare providers.

These findings necessitate a shift in diabetic foot education from traditional knowledge-focused approaches to targeted and detailed interventions that effectively address attitudinal barriers alongside knowledge provision. Healthcare providers should prioritize education on preventive topics over physiological mechanisms, implement formal, structured programs rather than informal information provision, and tailor strategies based on demographic factors, especially targeting younger patients and those with lower educational levels. By strengthening the critical pathway from preventive knowledge to treatment adherence and hygiene practices, physicians will have the ability and responsibility to optimize resource allocation and reduce the burden of diabetic foot complications.

## Supplementary Material



## Data Availability

The data supporting the findings of this study are available from the corresponding author upon reasonable request.

## Personal thanks

The author gratefully acknowledges Klinika Research Organization for their expertise in conducting the statistical analysis and providing methodological support for the structural equation modeling.
