# Supplementary material for: Modeling heterogeneity of diabetic foot self-care behaviors in Al Qassim Region in Saudi Arabia
Source: J Med Life. 2025 Sep;18(9):893–903. doi: 10.25122/jml-2025-0137 (PMC12577787; doi:10.25122/jml-2025-0137)
Supplement: Supplementary file 1 [file JMedLife-18-893-s001.pdf]

Supplementary Table 1. Direct, indirect, and total effects in knowledge-attitude-practice pathways

Structural Path	Standardized Coefficient (β)	SE	t-value	*P* value	95% CI (Bootstrap)
**Direct Effects**					
**Knowledge to Attitude**					
Physiological Knowledge to Treatment Adherence Attitudes	0.175	0.049	3.571	<0.001	[0.082, 0.268]
Physiological Knowledge to Preventive Behavior Attitudes	0.142	0.051	2.784	0.005	[0.046, 0.238]
Complication Knowledge to Treatment Adherence Attitudes	0.183	0.048	3.813	<0.001	[0.091, 0.275]
Complication Knowledge to Preventive Behavior Attitudes	0.165	0.050	3.300	0.001	[0.069, 0.261]
Preventive Knowledge to Treatment Adherence Attitudes	0.497	0.042	11.833	<0.001	[0.417, 0.577]
Preventive Knowledge to Preventive Behavior Attitudes	0.452	0.044	10.273	<0.001	[0.368, 0.536]
**Attitude to Practice**					
Treatment Adherence Attitudes to Hygiene Practices	0.316	0.046	6.870	<0.001	[0.228, 0.404]
Treatment Adherence Attitudes to Inspection Practices	0.284	0.047	6.043	<0.001	[0.194, 0.374]
Treatment Adherence Attitudes to Protective Practices	0.302	0.047	6.426	<0.001	[0.212, 0.392]
Treatment Adherence Attitudes to Healthcare Utilization	0.275	0.048	5.729	<0.001	[0.183, 0.367]
Preventive Behavior Attitudes to Hygiene Practices	0.275	0.046	5.978	<0.001	[0.187, 0.363]
Preventive Behavior Attitudes to Inspection Practices	0.241	0.047	5.128	<0.001	[0.151, 0.331]
Preventive Behavior Attitudes to Protective Practices	0.256	0.047	5.447	<0.001	[0.166, 0.346]
Preventive Behavior Attitudes to Healthcare Utilization	0.232	0.048	4.833	<0.001	[0.140, 0.324]
**Knowledge to Practice (Direct Paths)**					
Physiological Knowledge to Hygiene Practices	0.038	0.049	0.776	0.438	[-0.056, 0.132]
Physiological Knowledge to Inspection Practices	0.035	0.050	0.700	0.484	[-0.061, 0.131]
Physiological Knowledge to Protective Practices	0.041	0.049	0.837	0.403	[-0.053, 0.135]
Physiological Knowledge to Healthcare Utilization	0.031	0.050	0.620	0.535	[-0.065, 0.127]
Complication Knowledge to Hygiene Practices	0.079	0.048	1.646	0.100	[-0.013, 0.171]
Complication Knowledge to Inspection Practices	0.083	0.049	1.694	0.090	[-0.011, 0.177]
Complication Knowledge to Protective Practices	0.101	0.048	2.104	0.035	[0.009, 0.193]
Complication Knowledge to Healthcare Utilization	0.089	0.049	1.816	0.069	[-0.005, 0.183]
Preventive Knowledge to Hygiene Practices	0.214	0.051	4.196	<0.001	[0.116, 0.312]
Preventive Knowledge to Inspection Practices	0.163	0.052	3.135	0.002	[0.063, 0.263]
Preventive Knowledge to Protective Practices	0.175	0.051	3.431	0.001	[0.077, 0.273]
Preventive Knowledge to Healthcare Utilization	0.152	0.052	2.923	0.003	[0.052, 0.252]
**Indirect Effects**					
**Knowledge through Attitude to Practice**					
Physiological Knowledge through Attitudes to Hygiene Practices	0.094	0.020	4.700	<0.001	[0.058, 0.130]
Physiological Knowledge through Attitudes to Inspection Practices	0.083	0.018	4.611	<0.001	[0.050, 0.116]
Physiological Knowledge through Attitudes to Protective Practices	0.089	0.019	4.684	<0.001	[0.054, 0.124]
Physiological Knowledge through Attitudes to Healthcare Utilization	0.079	0.017	4.647	<0.001	[0.048, 0.110]

© 2025 by the authors. This article is an open access article distributed under the terms and conditions of the Creative Commons Attribution (CC BY 4.0) license.

Supplementary Table 1. Continued. Direct, indirect, and total effects in knowledge-attitude-practice pathways

Structural Path	Standardized Coefficient (β)	SE	t-value	*P* value	95% CI (Bootstrap)
Complication Knowledge through Attitudes to Hygiene Practices	0.100	0.021	4.762	<0.001	[0.062, 0.138]
Complication Knowledge through Attitudes to Inspection Practices	0.089	0.019	4.684	<0.001	[0.054, 0.124]
Complication Knowledge through Attitudes to Protective Practices	0.095	0.020	4.750	<0.001	[0.059, 0.131]
Complication Knowledge through Attitudes to Healthcare Utilization	0.084	0.018	4.667	<0.001	[0.051, 0.117]
Preventive Knowledge through Attitudes to Hygiene Practices	0.268	0.030	8.933	<0.001	[0.215, 0.321]
Preventive Knowledge through Attitudes to Inspection Practices	0.238	0.028	8.500	<0.001	[0.187, 0.289]
Preventive Knowledge through Attitudes to Protective Practices	0.253	0.029	8.724	<0.001	[0.199, 0.307]
Preventive Knowledge through Attitudes to Healthcare Utilization	0.226	0.027	8.370	<0.001	[0.176, 0.276]
**Total Effects**					
**Knowledge to Practice (Direct + Indirect)**					
Physiological Knowledge to Hygiene Practices	0.132	0.047	2.809	0.005	[0.044, 0.220]
Physiological Knowledge to Inspection Practices	0.118	0.048	2.458	0.014	[0.028, 0.208]
Physiological Knowledge to Protective Practices	0.130	0.047	2.766	0.006	[0.042, 0.218]
Physiological Knowledge to Healthcare Utilization	0.110	0.048	2.292	0.022	[0.020, 0.200]
Complication Knowledge to Hygiene Practices	0.179	0.046	3.891	<0.001	[0.091, 0.267]
Complication Knowledge to Inspection Practices	0.172	0.047	3.660	<0.001	[0.082, 0.262]
Complication Knowledge to Protective Practices	0.196	0.046	4.261	<0.001	[0.108, 0.284]
Complication Knowledge to Healthcare Utilization	0.173	0.047	3.681	<0.001	[0.083, 0.263]
Preventive Knowledge to Hygiene Practices	0.482	0.043	11.209	<0.001	[0.400, 0.564]
Preventive Knowledge to Inspection Practices	0.401	0.045	8.911	<0.001	[0.315, 0.487]
Preventive Knowledge to Protective Practices	0.428	0.044	9.727	<0.001	[0.344, 0.512]
Preventive Knowledge to Healthcare Utilization	0.378	0.045	8.400	<0.001	[0.292, 0.464]
**Model Fit and Variance Explained**					
**Model Fit Indices (Partial Mediation Model)**					
Comparative Fit Index (CFI)	0.938				
Tucker-Lewis Index (TLI)	0.929				
RMSEA (90% CI)	0.049 [0.045, 0.053]				
χ^2 (df)	879.56 (459)				
**Model Comparison (vs. alternatives)**					
$\Delta\chi^2$ (Partial vs. Full Mediation)	115.58 (10)				
$\Delta\chi^2$ (Partial vs. Direct Effects Only)	368.12 (12)				
**Variance Explained (R^2)**					
Treatment Adherence Attitudes	0.521 [0.459, 0.583]				
Preventive Behavior Attitudes	0.438 [0.374, 0.502]				
Hygiene Practices	0.417 [0.353, 0.481]				
Inspection Practices	0.354 [0.290, 0.418]				

Notes and Abbreviations: * Standardized coefficients (β) reported for the partial mediation model. Effect sizes: Small (β ≈ 0.10), Medium (β ≈ 0.30), Large (β ≈ 0.50). CI = confidence interval (95% bootstrap, 5000 samples); SE = standard error; RMSEA = Root Mean Square Error of Approximation; CFI = Comparative Fit Index; TLI = Tucker-Lewis Index. * *p*-value < 0.05 (or as indicated by '<0.001'). Model comparison based on $\Delta\chi^2$ (significant difference favors more complex model if *p*-value < 0.05).

© 2025 by the authors. This article is an open access article distributed under the terms and conditions of the Creative Commons Attribution (CC BY 4.0) license.

Supplementary Table 2. Differential knowledge-practice pathways by demographic and clinical characteristics

Pathway	Overall Sample (*n* = 647)	By education level		By diabetes duration		By healthcare education		By age group
		Lower education (*n* = 169)	Higher education (*n* = 478)	Short duration (*n* = 144)	Long duration (*n* = 97)	Received (*n* = 327)	Not received (*n* = 320)	Younger (<40, *n* = 312)
**Knowledge to Attitude Pathways**								
Physiological Knowledge to Treatment Adherence	0.175 (0.082, 0.268)*	0.163 (0.048, 0.278)*	0.178 (0.075, 0.281)*	0.151 (0.023, 0.279)*	0.246 (0.097, 0.395)*	0.232 (0.115, 0.349)*	0.121 (0.012, 0.230)*	0.157 (0.043, 0.271)*
Complication Knowledge to Treatment Adherence	0.183 (0.091, 0.275)*	0.145 (0.033, 0.257)*	0.197 (0.095, 0.299)*	0.139 (0.015, 0.263)*	0.271 (0.126, 0.416)*	0.243 (0.127, 0.359)*	0.118 (0.011, 0.225)*	0.142 (0.032, 0.252)*
Preventive Knowledge to Treatment Adherence	0.497 (0.417, 0.577)*	0.462 (0.354, 0.570)*	0.512 (0.422, 0.602)*	0.426 (0.309, 0.543)*	0.583 (0.459, 0.707)*	0.583 (0.486, 0.680)*	0.413 (0.315, 0.511)*	0.431 (0.325, 0.537)*
Preventive Knowledge to Preventive Behavior	0.452 (0.368, 0.536)*	0.415 (0.305, 0.525)*	0.467 (0.373, 0.561)*	0.384 (0.265, 0.503)*	0.535 (0.407, 0.663)*	0.541 (0.440, 0.642)*	0.372 (0.270, 0.474)*	0.395 (0.287, 0.503)*
**Attitude to Practice Pathways**								
Treatment Adherence Attitudes to Hygiene Practices	0.316 (0.228, 0.404)*	0.287 (0.154, 0.420)*	0.327 (0.231, 0.423)*	0.276 (0.135, 0.417)*	0.371 (0.233, 0.509)*	0.375 (0.269, 0.481)*	0.254 (0.142, 0.366)*	0.298 (0.181, 0.415)*
Treatment Adherence Attitudes to Inspection Practices	0.284 (0.194, 0.374)*	0.241 (0.106, 0.376)*	0.301 (0.203, 0.399)*	0.243 (0.099, 0.387)*	0.348 (0.207, 0.489)*	0.349 (0.239, 0.459)*	0.216 (0.102, 0.330)*	0.262 (0.144, 0.380)*
Treatment Adherence Attitudes to Protective Practices	0.302 (0.212, 0.392)*	0.268 (0.134, 0.402)*	0.315 (0.217, 0.413)*	0.257 (0.114, 0.400)*	0.362 (0.222, 0.502)*	0.363 (0.255, 0.471)*	0.235 (0.122, 0.348)*	0.271 (0.153, 0.389)*
Treatment Adherence Attitudes to Healthcare Utilization	0.275 (0.183, 0.367)*	0.224 (0.089, 0.359)*	0.294 (0.194, 0.394)*	0.231 (0.087, 0.375)*	0.341 (0.199, 0.483)*	0.342 (0.232, 0.452)*	0.198 (0.084, 0.312)*	0.243 (0.123, 0.363)*
Preventive Behavior Attitudes to Hygiene Practices	0.275 (0.187, 0.363)*	0.235 (0.102, 0.368)*	0.289 (0.195, 0.383)*	0.226 (0.085, 0.367)*	0.338 (0.201, 0.475)*	0.328 (0.224, 0.432)*	0.219 (0.109, 0.329)*	0.241 (0.127, 0.355)*
Preventive Behavior Attitudes to Inspection Practices	0.241 (0.151, 0.331)*	0.196 (0.062, 0.330)*	0.258 (0.162, 0.354)*	0.195 (0.053, 0.337)*	0.308 (0.169, 0.447)*	0.295 (0.187, 0.403)*	0.183 (0.071, 0.295)*	0.208 (0.093, 0.323)*
**Direct Knowledge to Practice Pathways**								
Physiological Knowledge to Hygiene Practices	0.038 (-0.056, 0.132)	0.021 (-0.118, 0.160)	0.042 (-0.062, 0.146)	0.018 (-0.129, 0.165)	0.064 (-0.079, 0.207)	0.054 (-0.064, 0.172)	0.024 (-0.085, 0.133)	0.026 (-0.089, 0.141)
Complication Knowledge to Protective Practices	0.101 (0.009, 0.193)*	0.063 (-0.072, 0.198)	0.114 (0.012, 0.216)*	0.075 (-0.068, 0.218)	0.143 (0.002, 0.284)*	0.131 (0.015, 0.247)*	0.067 (-0.039, 0.173)	0.078 (-0.035, 0.191)
Preventive Knowledge to Hygiene Practices	0.214 (0.116, 0.312)*	0.172 (0.033, 0.311)*	0.231 (0.125, 0.337)*	0.158 (0.015, 0.301)*	0.285 (0.137, 0.433)*	0.276 (0.154, 0.398)*	0.148 (0.036, 0.260)*	0.171 (0.053, 0.289)*
Preventive Knowledge to Inspection Practices	0.163 (0.063, 0.263)*	0.119 (-0.020, 0.258)	0.178 (0.070, 0.286)*	0.117 (-0.028, 0.262)	0.242 (0.092, 0.392)*	0.233 (0.109, 0.357)*	0.093 (-0.021, 0.207)	0.127 (0.007, 0.247)*

© 2025 by the authors. This article is an open access article distributed under the terms and conditions of the Creative Commons Attribution (CC BY 4.0) license.

Supplementary Table 2. Continued. Differential knowledge-practice pathways by demographic and clinical characteristics

Pathway	Overall Sample (n = 647)	By education level		By diabetes duration		By healthcare education		By age group
		Lower education (n = 169)	Higher education (n = 478)	Short duration (n = 144)	Long duration (n = 97)	Received (n = 327)	Not received (n = 320)	Younger (<40, n = 312)
**Indirect Effects (Selected Key Pathways)**								
Preventive Knowledge → Attitudes → Hygiene Practices	0.268 (0.215, 0.321)*	0.228 (0.156, 0.300)*	0.284 (0.225, 0.343)*	0.218 (0.149, 0.287)*	0.335 (0.254, 0.416)*	0.341 (0.270, 0.412)*	0.191 (0.135, 0.247)*	0.219 (0.155, 0.283)*
Preventive Knowledge → Attitudes → Inspection Practices	0.238 (0.187, 0.289)*	0.195 (0.127, 0.263)*	0.252 (0.195, 0.309)*	0.188 (0.123, 0.253)*	0.304 (0.227, 0.381)*	0.308 (0.241, 0.375)*	0.163 (0.111, 0.215)*	0.189 (0.129, 0.249)*
**Chi-Square Difference Tests for Path Invariance**								
**Path Invariance by Education Level**	$\Delta\chi^2$ = 27.41, df = 12, p = 0.007	Significant invariance						
**Path Invariance by Diabetes Duration**				$\Delta\chi^2$ = 35.62, df = 12, p < 0.001	Significant invariance			
**Path Invariance by Healthcare Education**						$\Delta\chi^2$ = 41.83, df = 12, p < 0.001	Significant invariance	
**Path Invariance by Age Group**								$\Delta\chi^2$ = 29.07, df = 12, p = 0.004
**Measurement Invariance Tests**								
**By Education Level**	Model Type	χ^2	df	CFI	ΔCFI	RMSEA	ΔRMSEA	Decision
	Configural	1352.49	918	0.933	-	0.051	-	-
	Metric	1379.82	943	0.932	0.001	0.050	0.001	Supported
	Scalar	1425.18	970	0.928	0.004	0.052	0.002	Supported
**By Diabetes Duration**	Configural	1285.37	918	0.937	-	0.048	-	-
	Metric	1329.43	943	0.934	0.003	0.049	0.001	Supported
	Scalar	1417.61	970	0.924	0.010	0.054	0.005	Partially Supported
**By Healthcare Education**	Configural	1347.24	918	0.932	-	0.051	-	-
	Metric	1385.63	943	0.930	0.002	0.052	0.001	Supported
	Scalar	1462.79	970	0.921	0.009	0.055	0.003	Partially Supported
**By Age Group**	Configural	1321.58	918	0.934	-	0.050	-	-

Notes: * P-value < 0.05; CI = confidence interval; Higher Education = University education or higher; Lower Education = Secondary education or below; Short Duration = Less than six-years; Long Duration = More than ten-years. Measurement invariance criteria: ΔCFI ≤ 0.01 and ΔRMSEA ≤ 0.015 indicate invariance is supported.

© 2025 by the authors. This article is an open access article distributed under the terms and conditions of the Creative Commons Attribution (CC BY 4.0) license.

Supplementary Table 3. High-leverage knowledge components and practice outcomes implications for targeted interventions

Knowledge component	Total effect on practice	Knowledge-practice gap	Threshold score	Intervention metrics	Demographic considerations
**Ranked Knowledge Items by Total Effect on Practice Outcomes**					
KnowledgeQ12: Regular foot care prevents problems	0.482 (0.400, 0.564)	4.7%	4.2/5	API = 32.4%	High effectiveness across all demographics
KnowledgeQ10: Importance of wearing proper shoes	0.463 (0.381, 0.545)	13.1%	4.0/5	API = 27.8%	Most effective for patients with long diabetes duration
KnowledgeQ4: Diabetics more likely to develop gangrene	0.372 (0.290, 0.454)	9.7%	4.1/5	API = 24.3%	Greater effect in university-educated patients
KnowledgeQ3: Diabetes can lead to foot ulcers	0.358 (0.276, 0.440)	10.3%	4.0/5	API = 23.5%	More effective in patients who received education
KnowledgeQ5: Loss of sensation makes diabetics vulnerable	0.345 (0.263, 0.427)	11.7%	3.9/5	API = 21.9%	Greater effect in older patients (≥40 years)
KnowledgeQ6: Decreased blood flow makes diabetics prone to ulcers	0.329 (0.247, 0.411)	12.6%	3.8/5	API = 21.5%	Greater effect in patients with longer duration
KnowledgeQ11: Smoking increases foot complications	0.317 (0.235, 0.399)	18.1%	3.7/5	API = 19.6%	Similar effect across demographics
KnowledgeQ2: Diabetics may lose sensation in feet	0.267 (0.185, 0.349)	11.9%	3.8/5	API = 16.8%	Greater effect in females
KnowledgeQ8: High blood sugar damages blood vessels	0.251 (0.169, 0.333)	14.0%	3.7/5	API = 15.3%	Greater effect in higher educated patients
KnowledgeQ7: Diabetes causes changes in foot shape	0.234 (0.152, 0.316)	22.7%	3.6/5	API = 13.2%	Similar effect across demographics
KnowledgeQ1: Diabetics experience reduced blood flow	0.221 (0.139, 0.303)	14.1%	3.7/5	API = 12.8%	Similar effect across demographics
KnowledgeQ9: Signs of diabetic foot infection	0.193 (0.111, 0.275)	39.3%	3.5/5	API = 18.1%	Significantly greater effect in those who received education
**Knowledge Threshold Effects by Practice Domain**					
**Practice Domain**	**Knowledge Score Threshold**	**Probability of Good Practice Below Threshold**	**Probability of Good Practice Above Threshold**	**Odds Ratio**	**Demographic Modifiers**
Hygiene Practices	42/60 (70%)	36.7%	83.5%	8.7	Greater threshold effect in females
Inspection Practices	45/60 (75%)	28.3%	79.4%	9.8	Greater threshold effect in older patients
Protective Practices	40/60 (67%)	32.9%	78.7%	7.5	Greater threshold effect in educated patients
Healthcare Utilization	48/60 (80%)	24.6%	82.1%	13.9	Strongest threshold effect in those with long duration

© 2025 by the authors. This article is an open access article distributed under the terms and conditions of the Creative Commons Attribution (CC BY 4.0) license.

Supplementary Table 3. Continued. High-leverage knowledge components and practice outcomes implications for targeted interventions

**Knowledge-Practice Gaps by Demographic Segments**					
**Demographic Group**	**Overall Knowledge Score**	**Overall Practice Score**	**K-P Gap**	**Highest Gap Practice Domain**	**Gap-Specific Intervention Priority**
Age < 40 years	41.6/60	8.7/16	19.1%	Inspection Practices (31.4%)	Focus on using mirror to check soles
Age ≥ 40 years	46.8/60	10.4/16	14.2%	Healthcare Utilization (23.5%)	Focus on regular monitoring of blood sugar
Female	44.7/60	10.1/16	15.3%	Protective Practices (25.7%)	Focus on wearing diabetic socks
Male	43.9/60	9.2/16	18.5%	Inspection Practices (29.8%)	Focus on daily foot checks
University Education	45.8/60	10.3/16	14.8%	Healthcare Utilization (24.1%)	Focus on regular medical follow-up
Lower Education	40.3/60	7.8/16	23.5%	All Domains (>20%)	Comprehensive education needed
Healthcare Education Received	47.1/60	11.6/16	8.4%	Protective Practices (15.2%)	Reinforce protective behaviors
No Healthcare Education	41.5/60	7.6/16	24.9%	All Domains (>20%)	Focus on basic knowledge-practice links
Diabetes Duration > 10 years	46.9/60	10.8/16	13.1%	Healthcare Utilization (21.7%)	Focus on consistency in care
Diabetes Duration < 6 years	42.6/60	8.5/16	19.8%	Inspection Practices (32.3%)	Focus on establishing daily routines
**Attributable Practice Improvement Percentage (API) by Knowledge Component**					
**Knowledge Component**	**Current Knowledge Level (%)**	**Practice Level if 100% Knowledge (%)**	**API**	**Number Needed to Educate**	**Population Level Impact**
Preventive Knowledge (All 3 items)	82.3%	93.7%	11.4%	8.8	High impact across all demographics
Complication Knowledge (All 5 items)	85.2%	92.1%	6.9%	14.5	Medium impact, especially for uncontrolled diabetes
Physiological Knowledge (All 4 items)	86.1%	90.3%	4.2%	23.8	Lower impact, foundation for understanding

© 2025 by the authors. This article is an open access article distributed under the terms and conditions of the Creative Commons Attribution (CC BY 4.0) license.

Supplementary Table 3. Continued. High-leverage knowledge components and practice outcomes implications for targeted interventions

**Simulation of Practice Improvement with Targeted Knowledge Enhancement**					
**Intervention Type**	**Baseline Practice Level**	**Expected Practice Level**	**Improvement**	**Resources Required**	**Implementation Considerations**
Knowledge Deficit Identification	63.7%	68.9%	5.2%	Minimal	Screening questionnaire in clinic
Focus on Gaps in Protective Practices	63.7%	71.5%	7.8%	Low	Educational materials on foot protection
Pathway-Targeted Education	63.7%	74.8%	11.1%	Moderate	Training on critical knowledge-attitude-practice links
Demographic-Tailored Intervention	63.7%	78.6%	14.9%	Moderate-High	Customized educational programs by segment
Comprehensive KAP Intervention	63.7%	82.3%	18.6%	High	Full program with follow-up reinforcement
**Critical Mediation Pathways and Intervention Points**					
**Mediation Pathway**	**Mediation Proportion**	**Direct Effect Proportion**	**Critical Intervention Point**	**Intervention Type**	**Expected Improvement**
Preventive Knowledge → Treatment Adherence → Hygiene	55.6%	44.4%	Attitude formation	Focus on importance of adherence	10.4%
Complication Knowledge → Preventive Behavior → Protection	48.5%	51.5%	Both knowledge and attitude	Dual-focus intervention	8.7%
Physiological Knowledge → Multiple Attitudes → Inspection	70.3%	29.7%	Primarily attitudes	Motivational enhancement	6.3%
All Knowledge Types → Healthcare Utilization	59.8%	40.2%	Primarily attitudes	Action planning for healthcare visits	9.2%

Notes and Abbreviations: * *P*-value < 0.05; API = Attributable Practice Improvement percentage (estimated increase in practice adherence if knowledge gap were eliminated); K-P Gap = Knowledge-Practice gap (difference between standardized knowledge and practice scores); Number Needed to Educate = Number of patients who need to receive focused education to gain one additional patient with good practice.

© 2025 by the authors. This article is an open access article distributed under the terms and conditions of the Creative Commons Attribution (CC BY 4.0) license.
